# Health workers’ perspectives on informed consent for caesarean section in Southern Malawi

**DOI:** 10.1186/s12910-021-00584-9

**Published:** 2021-03-29

**Authors:** Wouter Bakker, Siem Zethof, Felix Nansongole, Kelvin Kilowe, Jos van Roosmalen, Thomas van den Akker

**Affiliations:** 1 St. Luke’s Hospital, Malosa, Malawi; 2 Department of Obstetrics and Gynaecology, Leiden University Medical Centre, Leiden, The Netherlands; 3 Faculty of Science, Athena Institute, VU University Amsterdam, Amsterdam, The Netherlands

## Abstract

**Objective:**

Informed consent is a prerequisite for caesarean section, the commonest surgical procedure in low- and middle-income settings, but not always acquired to an appropriate extent. Exploring perceptions of health care workers may aid in improving clinical practice around informed consent. We aim to explore health workers’ beliefs and experiences related to principles and practice of informed consent.

**Methods:**

Qualitative study conducted between January and June 2018 in a rural 150-bed mission hospital in Southern Malawi. Clinical observations, semi-structured interviews and a focus group discussion were used to collect data. Participants were 22 clincal officers, nurse-midwives and midwifery students involved in maternity care. Data were analysed to identify themes and construct an analytical framework.

**Results:**

Definition and purpose of informed consent revolved around providing information, respecting women’s autonomy and achieving legal protection. Due to fear of blame and litigation, health workers preferred written consent. Written consent requires active participation by the consenting individual and was perceived to transfer liability to that person. A woman’s refusal to provide written informed consent may pose a dilemma for the health worker between doing good and respecting autonomy. To prevent such refusal, health workers said to only partially disclose surgical risks in order to minimize women's anxiety. Commonly perceived barriers to obtain a fully informed consent were labour pains, language barriers, women’s lack of education and their dependency on others to make decisions.

**Conclusions:**

Health workers are familiar with the principles around informed consent and aware of its advantages, but fear of blame and litigation, partial disclosure of risks and barriers to communication hamper the process of obtaining informed consent. Findings can be used to develop interventions to improve the informed consent process.

**Supplementary Information:**

The online version contains supplementary material available at 10.1186/s12910-021-00584-9.

## Background

Acquiring informed consent is considered essential in surgical practice, but clinical urgency such as in emergency caesarean section may hamper its practice [1–3]. Valid informed consent implies that the patient accepts an intervention willingly after comprehending adequate information about associated risks and benefits [1]. It derives from a patient’s fundamental right to self-determination regarding medical decisions and generally comprises a legal obligation across all settings [3]. Informed consent, however, is recognized as more than just a legal requirement. It creates partnership between patients and health workers aimed at improving quality of care, outcomes and patients’ satisfaction [3]. For all these reasons, obtaining informed consent should also be commonplace prior to caesarean section, the most frequently performed surgical intervention worldwide [4].

Although embedded in international and national codes and standards, studies and reports have shown the informed consent process for caesarean section lacking adequate discussion of surgical risk and being timed inappropriately [3, 5–13]. Women may be asked to provide consent while already on the theatre table or after surgery [7, 11]. Some perceived lack of communication by health workers with regard to medical decisions during birth [12, 13]. Many women have unplanned caesarean sections, in which labour pain and urgency may compromise quality of the consent process [1, 2]. Providing insufficient information and disregarding treatment preferences of women may create a sense among women and their partners that care is not in their best interest or that of their future children [12]. Non-consented care constitutes a form of ‘disrespect and abuse’ in maternity care and a barrier for women to seek skilled professional care in health facilities, where shared decision-making could reduce disrespect and abuse [14, 15].

Views differ between health workers and women regarding type and extent of preoperative information that had to be given, including which surgical risks [16, 17]. Such differences might lead to a consent process not living up to the expectations of women or health workers. In order to improve informed consent practice, it is therefore important to determine quality as perceived by women and health workers. Few studies have specifically focussed on health workers’ perspectives of informed consent and their views have been underrepresented in previous studies regarding decision-making in caesarean section or surgery at large [18, 19]. This study, conducted in a small rural hospital in southern Malawi, explores perceptions of health workers with regard to the informed consent process for caesarean section. Findings may be helpful in concurrent intervention development to improve care around caesarean section.

## Methods

This qualitative study was conducted between January 1st and June 1st 2018. The study is based on a social constructivist's view that relies on the complexity of participants’ perceptions to develop a theory around a particular subject [20]. This report applies the consolidated criteria for reporting qualitative research (COREQ) [21].

## Research team

As primary investigators, WB and SZ were involved in conception, study design and data analysis. WB is a medical doctor in Global Health and Tropical Medicine (MD GHTM), trained in the Netherlands. He has experience with obtaining surgical informed consent, both in the Netherlands and Malawi. At the start of this study, he had been working in the study facility for one-and-a-half year as medical officer involved in maternity care, working closely with and supervising participants. WB was clinical head of the maternity department, but not part of the hospital management at the time of the study and therefore had no influence or role in staff hiring or conducting performance reviews with any of the participants. SZ is a Bachelor of Science in Medicine from the Netherlands without clinical experience at the time of data collection, but had gained substantial knowledge with the concept informed consent during his training in the Netherlands. He arrived onsite 1 month before the start of the study without prior relationship to the location or study participants. Participants were made aware of his background and role as a researcher prior to any interactions with him. During observations of clinical practice, SZ developed professional relationships with most participants.

### Study setting

Malawi is a low-resource country in sub-Saharan Africa with a gross domestic product per capita of $390 in 2018 [22]. The study was performed in St. Luke’s Hospital, a rural mission hospital in Zomba District in Southern Malawi. The hospital supervises eight health centres in and around the district, of which two regularly refer obstetric emergencies to the hospital. The maternity department provides services free-of-charge and has an average of 200 births per month. The labour ward has five beds separated by curtains. Women are expected to bring cloths and blankets for use during labour, except in case of surgery when hospital linen is provided. Options for pain relief in this setting are limited: epidural analgesia and nitrous oxygen are not available and pethidine is only administered during the early stages of labour. A guardian, usually a senior female family member, is permitted as birth partner in the labour ward. Guardians serve a very important purpose in Malawian hospitals and all patients are expected to be accompanied by at least one guardian to assist in care tasks for the duration of their stay.

The national caesarean section rate was 6% in 2016, but the facility rate was 17% (163 out of 938 births) during the study period with the majority (84%) being unplanned [8, 23]. The majority of caesarean sections in the study facility are performed under spinal anaesthesia. General anaesthesia, most often involving ketamine, is used in case of insufficient spinal anaesthesia or critical emergencies as fetal distress or severe maternal concerns. In Malawi, general anaesthesia were more common until relatively recently, with 60% of caesarean sections still performed under general anaesthesia in 2000 [24].

Official languages in Malawi are Chichewa and English. The population comprises different ethnic groups, Yao (speaking Chiyao) and Chewa (Chichewa) being the largest in this setting [25]. Staff generally communicate with patients in Chichewa, but the official language during handover and for medical documentation is English. Among women giving birth by caesarean section, one in five were not able to read Chichewa or English, and half solely attended primary school or had not received any formal education [8].

### Study participants

Maternity staff comprised of nurses, midwives, midwifery students, clinical officers and two Dutch expatriate MD GHTM. There is rapid turnover with newly qualified staff fulfilling most clinical positions, whereas experienced employees move to supervisory or managerial positions at district, regional or national levels. Midwives either have 2 years of training in general nursing followed by 1 year of midwifery in one of the 14 nursing colleges to become a nurse-midwife technician (NMT), or complete a 4-year Bachelor of Science degree in Nursing and Midwifery at the University of Malawi to become a registered nurse-midwife (RNM) [26]. Nurse-midwives and nursing and midwifery students perform a sizable portion of patient care and are a substantial proportion of the hospital workforce. Clinical officers are associate clinicians with a predominantly practical training of 4 years [27]. Additionally, some completed a 2-year Bachelor's programme in obstetrics and gynaecology. Standard clinical care and most surgical interventions in rural Malawian hospitals are performed by clinical officers [27]. MD GHTM are general medical doctors who, after their medical licensing examination, undergo an additional two-and-a-half year training program consisting of traineeships in obstetrics and gynaecology combined with either surgery or paediatrics, a 6-month internship in a low-resource setting and clinical courses on infectious diseases, public health and health systems [28, 29]. Using purposive sampling, health workers from different professional cadres and from the entire maternity department were included [30].

### Informed consent

Informed consent is embedded in section 19(5) of the Malawian Constitution [31]. The Medical Council of Malawi implemented the informed consent concept in their Code of Ethics and Professional Conduct as a required inquiry for patients before any surgical procedure [6]. Bioethics, including informed consent practice, has been taught in preservice training of nurses and medical doctors by the Centre for Bioethics in Eastern and Southern Africa since 2009 [32, 33]. The informed consent process around caesarean section involves a conversation between surgeon and woman. Formally, when women are under-age, unable or considered incapable to provide consent, the guardian is her spokesperson and may provide consent on her behalf [6]. Informally, guardians are involved more often, with the majority (76%) of consent forms signed by guardians, as seen in local audit. The caesarean section consent form states that technicalities of the procedure, indication for surgery and related risks are explained and understood, and that the form has to be signed by the woman or her guardian, as well as the surgeon (Additional file 1: Appendix 1).

### Data collection

Data were collected by SZ following triangulation of methods: clinical observations, semi-structured interviews and a focus group discussion (FGD). To understand study setting, work processes and cultural parameters, at first different elements of the maternity department were observed and field notes made [34]. Secondly, semi-structured interviews were held in English. These one-to-one interviews were conducted in a private location in proximity to the participants' workplace and recorded with consent using a digital voice recorder. An interview guide was developed for this study based on observations and prior studies of informed consent practices in sub-Saharan Africa [16, 35]. This interview guide has not been published elsewhere and is added to this manuscript as Additional file 2: Appendix 2. We wanted participants to reflect on their experiences and discuss their perceptions of the informed consent process around caesarean sections, its purpose and ethical background. The guide was used flexibly, allowing participants to take different directions during the interview. Slight adjustments to the guide were made after two pilot interviews, increasing use of open-ended questions. After completing the interviews, an FGD was organized to validate identified barriers. This FGD included several interview participants representing different cadres and departments.

### Data analysis

Data collection and analysis occurred simultaneously and data collection was stopped when data saturation was observed [36]. Interviews and FGD were transcribed verbatim and checked concurrently to exclude obvious mistakes. Thematic analysis followed the framework method [37]. Transcripts were coded with the interview guide as initial format, while identifying points of interest through the entire process. Similar codes were grouped into themes, which could be applied deductively to remaining transcripts and revised if necessary. Initial coding was done independently by both primary investigators as to compare codes. When disagreement occurred, codes were re-evaluated and redefined to cover transcripts more accurately. Intercoder agreement was also established throughout the coding process by constantly discussing candidate themes and cases contradicting these themes [38]. A final set of distinct themes made up the analytical framework in which remaining data could be charted to generate rich descriptions. Transcripts were coded using a tabular summary in Microsoft Word files as a codebook.

## Results

Twenty-two health workers participated in the study; 20 were interviewed individually. The FGD included four participants (two NMT, one RNM and one clinical officer). Two participants were interviewed individually and participated in the FGD. None of the approached health workers declined or withdrew. Interviews lasted 27 to 81 min with a median of 45 min.

Median age was 30 years (inter-quartile range (IQR) 24–35) and 13 (59.1%) were males (Table 1). Most participants were NMT (n = 9; 40.9%) and the median professional experience was 2 years (IQR 1–8). All participants were directly involved in maternity care, of whom 16 (73%) on a daily basis. All had practiced informed consent in caesarean sections on multiple accounts, from around 20 to over 300 times. Although participants suggested roles for both midwife and surgeon, during observations midwives were the ones providing most information, ensuring women to sign the consent forms. Participants preferred the surgeon to be involved in the consent process as well to confirm the indication and explain the procedure. When asked why this often did not happen, a clinical officer (interview 2) mentioned "*it depends on the mentality. We think preparations should be done by the nurse.*"

**Table 1:** Characteristics of interviewed health care workers

	N = 22
Gender (%)	
Male	13 (59.1)
Female	9 (40.9)
Median age (IQR)	30 (24–35)
Education (%)	
Midwifery student	4 (18.2)
Nurse-midwife technician	9 (40.9)
Registered nurse-midwife	2 (9.1)
Clinical officer	6 (27.3)
MD GHTM	1 (4.5)
Median years of working experience (IQR)	2 (1–8)
Current department (%)	
Labour ward	10 (45.5)
Postnatal ward	2 (9.1)
Antenatal clinic	2 (9.1)
Operating theatre	2 (9.1)
OTHER (OPD, female ward^a^)	6 (27.3)

*IQR* interquartile range, *MD GHTM* medical doctor global health and tropical medicine, *OPD* outpatient department

^a^Female ward: general medical and surgical ward for women

### Definition and function of informed consent

All health workers were familiar with the concept of informed consent and able to provide a definition. Definitions included sharing information and decision-making. Informed consent was understood as a means to obtain approval: the consenter gives permission for a proposed intervention, thereby accepting its benefits and risks. Consent was primarily considered to be of legal value. Other identified purposes of informed consent were patient education, respecting autonomy, acknowledging patient rights and improving the health worker-patient relationship (Table 2).

**Table 2:** Health care workers’ purpose of informed consent and what should be discussed

Purposes of informed consent (from frequent to less frequent)	Legal protection of health care worker Patient education, which: Reduces complications postoperatively Prepares psychologically and reduces anxiety Benefits subsequent pregnancies Respecting autonomy Respecting human/patient rights Improving woman—health care worker relationship
Topics to discuss (from frequent to less frequent)	Reason for caesarean section Complications of caesarean section Infection Haemorrhage/haemorrhagic shock Extended recovery time Injury bladder/ureter/bowel Maternal death Leaving instruments in abdomen Hysterectomy Feeling pain during surgery Procedure Use of anaesthetics Use of blood products Limited number of children due to a maximum of three CS Need to give birth in hospital in subsequent pregnancies

Three major themes illustrated challenges to practice informed consent in line with its mentioned definition: fear of blame and litigation, partial disclosure of risks and communication barriers. A diagram of the coding tree is shown in Additional file 3: Appendix 3.

### Fear of blame and litigation

Health workers expressed feeling accountable and sometimes criticised when involved in cases of maternal or perinatal death or severe morbidity. Potential critics were the woman's relatives, members of the public, fellow health workers, professional medical associations and politicians. Five participants expressed fear of losing their jobs if adverse events occurred during childbirth. Although none declared to have been involved in an obstetric legal case, legal repercussions were often mentioned. An RNM (interview 7) expressed feeling increased pressure whilst working in maternity, since "*obstetric issues do attract legal teams very often, as compared to general wards.*" One participant felt that fear of being blamed could lead to increased quality of care by improving use of protocols and ameliorating documentation, where others expressed that such fear could paradoxically lead to mistakes and cause health workers to shun away from taking challenging clinical decisions. Occasionally, hesitation to be involved in such situations, irrespective of staff cadre, was affirmed by clinical observations.

"They come only when mistakes are made. That will install elements of fear to health workers. So sometimes when you are working under fear, you also make blunders. So, what happens is that most of us, or most health workers, nurses, work with that element of fear." (Clinical officer, FGD)

In a challenging obstetric situation, obtaining written consent for caesarean section was said to alleviate fear of blame for adverse obstetric outcomes, regardless of mode of birth. Participants considered written consent as an acknowledgement by women of being informed and agreeing to the proposed surgical intervention. Responsibility would be shared and in case complications would arise, health workers would not feel liable to the same extent. According to an NMT (interview 15), providing informed consent gives the woman a choice, "*and whenever she is making the decision, she will know that she is accountable and not to blame someone else.*" Transfer of liability was mentioned as being protective in legal trials.

"They may see you in court and if the lady has signed consent; that will serve as an explanation. Then it is going to back you up, saying; I explained this procedure to the woman and she agreed and gave consent, saying that we should go on and she signed. That means you are safe on your part." (Clinical officer, interview 17)

Whereas obtaining written consent alleviated fear, failing to do so would aggravate this. Written consent was considered obligatory for all caesarean sections. Performing caesarean section without such consent would render the surgeon liable. Although refusing caesarean section was stated to be uncommon, half of the interviewees had experienced such a situation. This was thought to be best handled by documenting the woman’s refusal to undergo surgery and having her affix her signature to this document. Participants felt that when women signed for refusal, this would protect them equally against litigation as when women signed for caesarean section. During the FGD, participants agreed that practicing informed consent purely to obtain written approval was seen as disadvantageous, as a clinical officer described:

"The reason to do informed consent is because of fear of a legal pursue when not done… To me that is not right. It should be because the patient should be informed. She should know why she is taken for what procedure, or what intervention, right? The issue of legal thing should not mean anything." (Clinical officer, FGD)

### Partial disclosure of risks

Most caesarean sections were unplanned and performed during active labour, at a time health workers established that caesarean section would be the best option for woman and child. When asked whether women were able to refuse, many health workers believed the need to provide optimal care should overrule her option of refusal. Only four participants, who received additional training at the University of Malawi and had more than average work experience, considered women's preferences over the need to do good, even if her preference was not aligned with their idea of optimal obstetric care. However, in case of obstetric emergencies such as cord prolapse, eclampsia, antepartum haemorrhage or ruptured uterus, all participants agreed on the need for immediate action, overruling women’s autonomy and omitting the option for women to refuse surgery, as consultation time was thought to jeopardize care. In some cases, women would be in critical condition and judged to lack capacity to consent, in which instance written consent was obtained from guardians. During the study period, four out of 160 caesarean sections were performed without written consent in the surgical files, all attributed to obstetric emergencies.

"For emergencies we do not spend much time. We just explain to them the reason why we are taking them for CS [caesarean section]. Usually guardians make an informed decision for them. And they sign. Maybe the patient, if she is strong enough, but mostly guardians were the ones who were responsible for giving consent." (RNM interview 7)

The dilemma created by women refusing caesarean sections led to seemingly contradictory statements: interviewees insisted on caesarean section, but would not go to the operating room without the woman's consent (Table 3). Especially student nurses made conflicting statements. A clinical officer (interview 1) captured the internal conflict of wanting to provide adequate care while also taking patient's autonomy into account: "*Currently there is the responsibility of accepting the consequences [of a treatment decision], but if I was given the chance to edit the patient's responsibilities, I would write among the points that the patient has the responsibility of accepting the important treatments.*" He reasoned that health workers act in the patients best interest and that it is very rare for health workers to intentionally harm their patients.

**Table 3:** Contradictory statements in interviews: need for intervention versus need for consent

Interview number	Need for intervention	Need for consent
7, RNM	"But we can weigh the benefits and the risks. Cause the woman will not deliver, we should operate on the patient."	"She has refused. And she has refused to sign the consent. You cannot force her."
9, student midwife	"Giving good healthcare despite what the patient thinks. Because we are the one who can see the danger which the woman can have. So, I think I'll leave the autonomy aside and then concentrate on the health of my patient"	"We can't just take the patient to the theatre when she says no."
11, student midwife	We are going to take the patient to theatre. We can't do otherwise	But we can't just take the patient to the theatre when she says no
12, student midwife	It is impossible to say no	You can't force

Health workers explained that sometimes, in order to convince a woman that caesarean section was the preferred management option, they chose to emphasize certain parts of the information. Some would present the advantages clearly, whilst downplaying risks out of fear to induce anxiety. Others would emphasize risk of continuing vaginal birth in order to increase willingness to undergo surgical treatment. Participants used different terms for this practice: ‘deviation’, ‘imposition’, ‘coercion’ or ‘blanket consent’. A clinical officer (interview 20) had recently encountered a woman who declined caesarean section after a thorough risk discussion, resulting in feelings that "*after explaining all risks, some will be afraid and will be troublesome to go for CS.*" Another clinical officer (interview 2) shared this notion, stating that: "*if you see that the patient is very anxious about CS and you feel like that by telling her every detail, she might back off … then I would not tell her everything.*"

Although used commonly, incomplete risk discussion was seen as poor clinical practice. Other ways to deal with women's anxiety for caesarean section were mentioned, such as weighing risks and benefits and discussing management of possible complications. An experienced clinical officer suggested that when the woman is not informed adequately, provided consent is invalid and ineffective:

"There is something wrong with it [partial disclosure of risks]. It will affect you again. If something goes wrong during the procedure, you will be in the wrong. The most important thing is that they will understand it and teach the others. They will tell the others what happened. But if they do not understand you do CS on this one, no one will understand." (Clinical officer, interview 19)

FGD participants agreed that an informed consent process focussing mainly on obtaining written consent could lead to signing without actually being informed, thereby defying the purpose of the informed consent process.

### Communication barriers

Several barriers impairing communication during the informed consent process were mentioned, such as lack of knowledge and difference in language. Women with low levels of education were thought to have limited understanding of caesarean section. Some women had expressed fear of dying from (general) anaesthesia to health workers, believing they had been cursed if they failed to give birth vaginally. Health workers predominantly spoke Chichewa, while many women spoke Chiyao only, requiring translation by other staff or guardians. Consent forms were not available in Chiyao, so Yao women and guardians relied on verbal information. However, health workers found medical information difficult to express even in Chichewa, since this language was found to lack words covering medical terminology. Participants handled such barriers in different ways. One RNM (interview 7) explained:

"You tend to explain more to a patient that is looking smart, as compared to the one who is looking like she is coming from the village. With the assumption that maybe she cannot reason critically as compared to the [other] one."

On the contrary, some midwifery students made an additional effort by taking more time and using simple language. They asked women to paraphrase to check whether the information was understood.

"You never know who does not understand the condition. You generalize it. You treat everyone like they are able to understand it." (Midwifery student, interview 6)

Many health workers felt women expected them to make decisions, as women in general wards were thought to be unaware of their rights. A midwifery student (interview 11) pointed out that "*there is a problem of people not knowing they have the right to be informed about what is going to happen on their body. They think maybe someone has to intervene and help them.*" A clinical officer (interview 19) felt the same, saying "*they will accept because you are the doctor. They feel like their life is in your hands.*" One nurse pointed out that women could not easily access another hospital:

"We can also look at the economic status of those who come to the hospital. They are the people who like this hospital so much for reasons. So, they would say if I deny [informed consent for caesarean section] where will I go. Who will help me? They do not have much [choice]." (NMT, interview 5)

Often women would not be able to make decisions without guardians. Some women were thought to prefer health workers to discuss care with guardians instead. An NMT (interview 13) had a woman refusing to be counselled for caesarean section: "*She said 'Call my mother outside. Whatever my mother says; follow it.*" Others had to wait for husbands to give consent or family members consenting through the phone. Such dependency also applied to other decision-making than with regard to mode of birth, including the decision about sterilization during caesarean section:

"Because you find that they tell you; I have not discussed with my husband as to how many children we are going to bear. So, on her own she cannot choose. I can only give consent if my husband agrees." (Clinical officer, interview 17)

During observations, guardians would ask most questions during the informed consent process and aided in counselling women. For health workers, decisions made by women were valued over guardians' decisions, as long as these were regarded as rational.

Lastly, interviewees generally thought labour pains would negatively impact women's decision-making capacity. They felt reluctant to provide information, as this was seen as ineffective. They experienced that women would ask for and eagerly accept caesarean section while in labour, which was also witnessed during observations. Midwifery students working in labour ward (interviews 9 and 14) stated many women would "*cry*" for caesarean section, as "*they might have been in labour for a long time.*" Women were aware that anaesthetics used during surgery would relieve labour pains. Such cries for intervention were commonly regarded as implied consent:

"The decision is already made by those women. They just need the doctor to accept that." (Midwifery student, interview 14)

Even though participants sometimes doubted the validity of such implied consent, generally they would go ahead with the procedure.

## Discussion

Informed consent was a widely known concept among health workers and all participants were able to provide a clear definition. Provided definitions revolved around promoting decision-making by the woman and were in accordance with its bioethical definition [3]. Several limitations to application of this knowledge in clinical practice were noted; fear of blame and liability, use of partial disclosure of risks and prevailing thoughts of women's lack of competence to participate in decision-making. These limitations were perceived to reduce the quality of informed consent. They resemble common ethical dilemmas in relation to informed consent, as the conflict with the need to do good, transfer of liability to and capability of the patient [2].

Major finding was that obtaining written consent for caesarean section would alleviate fear of blame for adverse obstetric outcomes. This has previously been identified as reason for increasing caesarean section rates [18]. Among Kenyan obstetricians, 27% named fear of litigation in relation to a falling trend of vaginal birth after caesarean section [39]. A majority (67%) of obstetricians in the United Kingdom and Ireland cited fear of litigation as one of the main reasons for rising national caesarean section rates [40]. In a tertiary hospital in Dar es Salaam, obstetric residents perceived caesarean section as a safe procedure, protecting them from being blamed for poor perinatal outcome associated with vaginal birth [41]. Written consent probably plays a key role in this practice, as it may be considered a transfer of liability and safeguard from litigation [2, 3]. An obstetrician from the United States once noted: "You don’t get sued for doing a C-section. You get sued for not doing a C-section” [42]. It is noteworthy that written consent is not considered equal to valid consent: it proofs a consent process took place, but does not address its quality [1, 2].

**Fig. 1:**
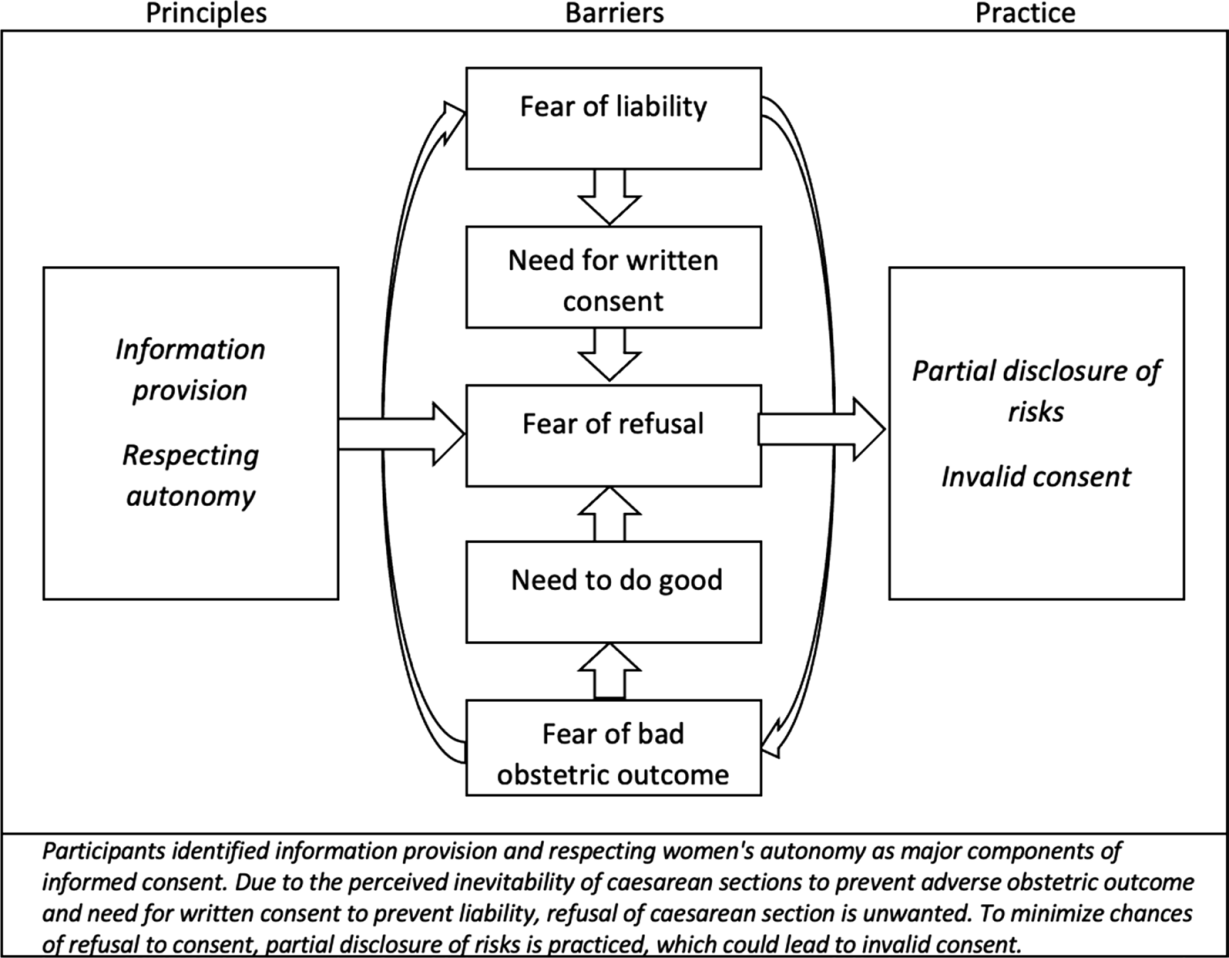
Analytical framework: principles into practice

Figure 1 shows the analytical framework in which the informed consent process is affected by fear of refusing surgery. Refusal would mean surgery without consent or adverse birth outcomes by continuing vaginal birth, both posing surgeons liable. Fear of refusal would lead to partial disclosure of risks, as health workers sometimes were afraid women would refuse when full disclosure was provided. Challenges in balancing comprehensive risk counselling and aggravating fear are very common dilemmas among obstetricians [17]. Assuming that discussing complications induces fear is not necessarily warranted, as women's reaction to risk disclosure vary from being overwhelmed to feeling in control [17, 43]. However, with a 1.3% chance of maternal death after caesarean section in low-resource settings, fear is not irrational [44]. From an ethical perspective, withholding information regarding procedure-related risks is incompatible with truth-telling and negates consent, while recommending treatment options does not violate informed consent per se [2]. Care should be taken not to compromise individuals’ own choice, so women can decide whether recommendations fit their beliefs and values [2, 3]. Current Malawian obstetrical guidelines do not include specific advice on how to ask consent for caesarean section [45].

Illiteracy, limited knowledge, labour pains and dependency on guardians were believed to complicate the consent process and have previously been identified as communication barriers in low- and high-income settings [35, 41, 46, 47]. Some participants argued that women lacked understanding of the given information and expected health workers to make decisions for them. This paternalistic view used to be the norm in global health care, but is decreasingly accepted [48]. While life-threatening emergencies justify paternalism, educational levels, labour pains or refusal of treatment do not [1, 3]. In case of low educational levels and language barriers, a structured informed consent process might still result in significant information recall [8, 47]. It was noted that paternalistic views often coexisted with knowledge of benefits of shared decision-making, indicating knowledge alone does not automatically result in corresponding practice.

## Limitations and strengths

Our study focussed only on perceptions of health workers and not of the women undergoing caesarean section, making the framework incomplete. Negative effects of identified barriers on women's roles in the consent process cannot be confirmed without including their perceptions. After all, it is decided by the woman’s expectations, not the medical profession, whether the consent process is regarded as acceptable for her to make a decision [3, 49]. Secondly, the team’s apparent roles as researchers, and additional role (WB) as supervisor of participants, might have resulted in social desirability during observations and interviews. This effect, however, was limited by having the interviews conducted solely by an external researcher who was not part of the clinical team (SZ) and anonymisation of the transcripts. Thirdly, due to similar cultural and professional backgrounds the primary investigators might have similar interpretations during analysis. Additionally, cultural background differed from that of the participants, which may have affected data interpretation. However, WB has been living and working in the study setting and has previous experience with qualitative research in Malawi [50]. Lastly, we did not conduct repeat interviews with the same participants to clarify statements made in their first interview [51].

Qualitative findings could serve intervention development and assure that interventions are well received by their intended audiences [52]. The current study may be an example, as the findings aided development and implementation of a consent form, guide and clinical training to improve informed consent for caesarean section [8]. In the study setting, risk recall by women who gave birth by caesarean section was unsatisfactory, which was partly attributed to partial disclosure of risks and its underlying rationale (Fig. 1). An informed consent training and complementary guide were developed accordingly, focussing on differences between written and valid consent, morbidity and mortality associated with caesarean section and discussion on increasing women's participation in case of labour pain, illiteracy and limited knowledge. Finally, a presentation joined by most hospital staff, led to a plenary discourse on these themes and may have been instrumental to accept the new consent form substantially. Themes may be generalizable to different populations, as 'fear of litigation' and 'transfer of liability' have found resonance in a meta-synthesis, and we found similarities with responses from health workers in Dar es Salaam [16, 18, 41]. Future research should re-examine our framework in different contexts and with different methodologies to confirm generalizability.

## Conclusions

The informed consent process around caesarean section was widely known and regarded as an essential part of obstetric practice. The process was challenged, however, by fear of blame for adverse obstetric outcomes and tended to focus on obtaining written consent to transfer liability. Health workers struggled with balancing women’s autonomy and providing optimal treatment, which sometimes caused them to disclose surgical risks only partly. They perceived the informed consent process being affected by labour pains, women’s dependency on others and limited levels of education. Understanding how principles of informed consent are affected by these barriers was of extreme importance for intervention development and implementation to strengthen the informed consent process.

## Supplementary Information

**Additional file 1.** Consent form for caesarean sections.

**Additional file 2.** Semi-structured interview guide.

**Additional file 3.** Coding tree.

**Additional file 4.** Consent form for participants.

## Data Availability

The datasets used and/or analysed during the current study are available from the corresponding author on reasonable request.

## Abbreviations

COREQ: Criteria for reporting qualitative research
FGD: Focus group discussion
IQR: Interquartile range
MD GHTM: Medical doctor global health and tropical medicine
NMT: Nurse-midwife technician
RNM: Registered nurse-midwife
