# Supplementary material for: Health workers’ perspectives on informed consent for caesarean section in Southern Malawi
Source: BMC Med Ethics. 2021 Mar 29;22:33. doi: 10.1186/s12910-021-00584-9 (PMC8008515; doi:10.1186/s12910-021-00584-9)
Supplement: Supplementary file 1 — Additional file 1. Consent form for caesarean sections. [file 12910_2021_584_MOESM1_ESM.docx]

**Appendix 1. Informed consent form for caesarean section**

| **DETAILS** | Patient name |  |  | **DECISION** | Date and time |  |
| --- | --- | --- | --- | --- | --- | --- |
|  | Next of kin |  |  |  | Made by |  |
|  | Contact details |  |  |  | Indication |  |

| **INFORMATION (BY SURGEON)** | Discuss the following topics with the patient  ⬜ Explained **INDICATION** for CS and **BENEFITS** of CS in current situation to the woman.  ⬜ Explained **PROCEDURE** of CS to the woman. *Including anaesthesia and possible use of blood products.*  ⬜ Explained **RISKS** of CS to the woman. *Infection, haemorrhage, recovery time, serious and rare complications*  ⬜ Explain **IMPLICATIONS FOR FUTURE PREGNANCIES.** *Hospital birth, trial of labour, risk of uterine rupture*  ⬜ Address **UNCERTAINTIES** and answer **QUESTIONS.**  ⬜ Gain **VERBAL CONSENT** from the woman. |
| --- | --- |
|  | I have explained the procedural nature and risks to the undersigned woman or person legally competent to give consent.  Surgeon: Signature: Date: |

| **CONSENT (BY PATIENT)** | I, the undersigned, hereby consent to the performance of, and understand the nature and risks of the procedure. The clinicians who perform the above may increase the reasonable scope thereof or carry out additional or alternative measures (including general anaesthesia) if considered necessary. I agree that a sample of my blood will be taken and tested for Hepatitis B and HIV should an incident of contamination of a health care worker by bodily fluids occur during the procedure. I grant consent to use blood and/or blood products if needed. |
| --- | --- |
|  | Woman/guardian name: Signature: Date:  Relationship to woman (if applicable): |
