# Supplementary material for: Health workers’ perspectives on informed consent for caesarean section in Southern Malawi
Source: BMC Med Ethics. 2021 Mar 29;22:33. doi: 10.1186/s12910-021-00584-9 (PMC8008515; doi:10.1186/s12910-021-00584-9)
Supplement: Supplementary file 2 — Additional file 2. Semi-structured interview guide. [file 12910_2021_584_MOESM2_ESM.docx]

# Interview guide: informed consent for caesarean section, health worker perspectives

**Introduction:** scope of study, length of interview, informed consent for current study.

**Characteristics:** gender, age, education, years of working experience, current occupation, number of caesarean sections involved in

**Primary and probing questions**

1. **Experiences with informed consent**

Could you describe the last informed consent process for caesarean section you were involved in?

- - - What were you trying to achieve?
    - What was the role of the woman?
    - Is informed consent mandatory in this case?

How would you consider your overall experience with informed consent?

Did any of the women ever refuse to be operated upon?

- How was the situation handled?
- If not, how would you hypothetically handle the situation?

1. **Definition of informed consent**

Could you provide your definition of informed consent?

According to you, what is the purpose of informed consent in caesarean sections?

According to you, what is the effect on women? And on health care workers?

1. **Informed consent in clinical practice**

How should informed consent for caesarean sections be practiced?

- - - What should be discussed?
    - Who should be involved in the discussion?
    - Who should provide written consent?
    - What is the role of the relatives/guardians?

Are there any barriers to informed consent practice?

Are there any situations where informed consent is inappropriate or unwarranted?

1. **Statements**

True or false. Elaborate!

- - Most women and their guardians do not understand the provided information.
  - Most women and their guardians expect the doctor to make a decision.
  - Most women are coerced into the health care worker’s option of choice.

1. **Conclusion**

Summarize and invite the interviewee to make adjustments and additions.

- Repeat the provided definition, advantages, disadvantages and challenges.
