# Supplementary material for: Health workers’ perspectives on informed consent for caesarean section in Southern Malawi
Source: BMC Med Ethics. 2021 Mar 29;22:33. doi: 10.1186/s12910-021-00584-9 (PMC8008515; doi:10.1186/s12910-021-00584-9)
Supplement: Supplementary file 3 — Additional file 3. Coding tree. [file 12910_2021_584_MOESM3_ESM.docx]

| **Appendix 2. Coding tree** | | |
| --- | --- | --- |
| Themes | Categories | Codes |
|  | | |
