# Supplementary material for: Health workers’ perspectives on informed consent for caesarean section in Southern Malawi
Source: BMC Med Ethics. 2021 Mar 29;22:33. doi: 10.1186/s12910-021-00584-9 (PMC8008515; doi:10.1186/s12910-021-00584-9)
Supplement: Supplementary file 4 — Additional file 4. Consent form for participants. [file 12910_2021_584_MOESM4_ESM.docx]

# Research: Health Worker Perspectives on Informed Consent for Caesarean Sections.

| Department | Obstetrics |
| --- | --- |
| Organization | Saint Luke's Hospital |
| Date |  |

Dear Sir, Madame,

The following interview will be conducted to gain insight in your perspectives on giving information to the patient and gaining her consent prior to CS. The interview will discuss personal experiences with informed consent, as well as thoughts about the information transfer, practical use of informed consent and ethical considerations.

- **The interviews are anonymous. The interviewee only states his/her function, age, gender, department and years of working experience.**
- **The interviews will be recorded and analyzed only by the interviewer.**
- **Comments may be used as quotes in the article. This, again, will be anonymous.**
- **The interview takes 30 minutes to 1 hour.**

By conducting interviews and analyzing them as one entity, the researcher tries to understand the process of informed consent and apply interventions were needed. This may improve the quality of care in Saint Luke's Hospital, but also may serve as an example for other health facilities.

This interview is not compulsory and will not affect your work in a negative way. The researcher may write during the interviews. The notations include observations and possible quotes.

**I hereby give my consent for participating in this study.**
